# A linked physiologically based pharmacokinetic model for hydroxychloroquine and metabolite desethylhydroxychloroquine in SARS‐CoV‐2(−)/(+) populations

**DOI:** 10.1111/cts.13527

**Published:** 2023-04-29

**Authors:** Claire Steinbronn, Yashpal S. Chhonker, Jenell Stewart, Hannah Leingang, Kate B. Heller, Meighan L. Krows, Michael Paasche‐Orlow, Anna Bershteyn, Helen C. Stankiewicz Karita, Vaidehi Agrawal, Miriam Laufer, Raphael Landovitz, Mark Wener, Daryl J. Murry, Christine Johnston, Ruanne V. Barnabas, Samuel L. M. Arnold

**Affiliations:** ^1^ Department of Pharmaceutics University of Washington Seattle Washington USA; ^2^ Department of Pharmacy Practice and Science University of Nebraska Medical Center Omaha Nebraska USA; ^3^ Division of Infectious Diseases Hennepin Healthcare Research Institute Minneapolis Minnesota USA; ^4^ Department of Medicine University of Minnesota Minneapolis Minnesota USA; ^5^ Department of Medicine University of Washington Seattle Washington USA; ^6^ Department of Global Health University of Washington Seattle Washington USA; ^7^ Department of Medicine Tufts Medical Center Boston Massachusetts USA; ^8^ Division of Primary Care Tufts Medical Center Boston Massachusetts USA; ^9^ Department of Population Health New York University Grossman School of Medicine New York New York USA; ^10^ Center for Vaccine Development and Global Health University of Maryland Baltimore Baltimore Maryland USA; ^11^ UCLA Center for Clinical AIDS Research and Education David Geffen School of Medicine at UCLA Los Angeles California USA; ^12^ Massachusetts General Hospital Boston Massachusetts USA; ^13^ Harvard Medical School Boston Massachusetts USA

## Abstract

Hydroxychloroquine (HCQ) is Food and Drug Administration (FDA)‐approved for malaria, systemic and chronic discoid lupus erythematosus, and rheumatoid arthritis. Because HCQ has a proposed multimodal mechanism of action and a well‐established safety profile, it is often investigated as a repurposed therapeutic for a range of indications. There is a large degree of uncertainty in HCQ pharmacokinetic (PK) parameters which complicates dose selection when investigating its use in new disease states. Complications with HCQ dose selection emerged as multiple clinical trials investigated HCQ as a potential therapeutic in the early stages of the COVID‐19 pandemic. In addition to uncertainty in baseline HCQ PK parameters, it was not clear if disease‐related consequences of SARS‐CoV‐2 infection/COVID‐19 would be expected to impact the PK of HCQ and its primary metabolite desethylhydroxychloroquine (DHCQ). To address the question whether SARS‐CoV‐2 infection/COVID‐19 impacted HCQ and DHCQ PK, dried blood spot samples were collected from SARS‐CoV‐2(−)/(+) participants administered HCQ. When a previously published physiologically based pharmacokinetic (PBPK) model was used to fit the data, the variability in exposure of HCQ and DHCQ was not adequately captured and DHCQ concentrations were overestimated. Improvements to the previous PBPK model were made by incorporating the known range of blood to plasma concentration ratios (B/P) for each compound, adjusting HCQ and DHCQ distribution settings, and optimizing DHCQ clearance. The final PBPK model adequately captured the HCQ and DHCQ concentrations observed in SARS‐CoV‐2(−)/(+)participants, and incorporating COVID‐19‐associated changes in cytochrome P450 activity did not further improve model performance for the SARS‐CoV‐2(+) population.

**Study Highlights**

**WHAT IS THE CURRENT KNOWLEDGE ON THE TOPIC?**

Physiologically based pharmacokinetic (PBPK) modeling is used to simulate drug absorption, distribution, metabolism, and excretion in virtual patient populations. Hydroxychloroquine (HCQ) and its metabolite desethylhydroxychloroquine (DHCQ) have demonstrated extensive pharmacokinetic (PK) variability which has not yet been well defined and was not captured by current PBPK models.

**WHAT QUESTION DID THIS STUDY ADDRESS?**

This study addresses how incorporating the variability in blood to plasma concentrations (B/P) for HCQ and DHCQ aids in explaining a portion of the observed PK variability. Additionally, it demonstrates that mild COVID‐19 is not expected to dramatically alter HCQ nor DHCQ PK, and it would take many participants to detect a meaningful change in the PK of each compound.

**WHAT DOES THIS STUDY ADD TO OUR KNOWLEDGE?**

Incorporating B/P variability into a PBPK model improves model performance for both HCQ and DHCQ. This is an important consideration for other drugs with extensive blood partitioning. By incorporating observed variability in this parameter, PBPK models can improve predictions of drug exposure.

**HOW MIGHT THIS CHANGE CLINICAL PHARMACOLOGY OR TRANSLATIONAL SCIENCE?**

This work demonstrates how B/P variability significantly impacts predictions of HCQ and DHCQ PK. B/P variability is important for consideration for other compounds as well and may be an important determinant of drug disposition. Furthermore, PBPK modeling software should incorporate user input of observed variability in this parameter.

## INTRODUCTION

Hydroxychloroquine (HCQ) is Food and Drug Administration (FDA)‐approved as a treatment and prophylaxis for malaria and for chronic management of chronic discoid and systemic lupus erythematosus and rheumatoid arthritis.^1^ While the exact mechanism of action is not clear for any indication, multiple inhibitory and immunomodulatory effects of HCQ have been proposed.^2^ Due to its perceived impact on a diverse array of physiological functions, HCQ has been investigated as a repurposed therapeutic for a range of indications including COVID‐19.^2^, ^3^ Opportunities to repurpose HCQ are further supported by its well‐characterized safety profile and its widespread global availability in part due to its designation as an essential medicine by the World Health Organization (WHO). Despite these positive attributes, lingering uncertainty about HCQ absorption, distribution, metabolism, and excretion (ADME) presents a hurdle when selecting a dosing regimen for any new indication.

Even with more than 60 years of use in the clinic, HCQ pharmacokinetics (PK) are still not sufficiently understood. The reported PK parameters for HCQ vary greatly and the sources of the observed variability have not yet been well characterized.^4^, ^5^, ^6^, ^7^ HCQ has a large and variable volume of distribution (*V*_D_) with reported values ranging from ~25,000 to 94,000 L.^4^ Studies conducted in rats demonstrated that HCQ has extensive and tissue‐dependent distribution that did not reach distribution equilibrium despite 7 months of daily HCQ dosing.^8^, ^9^, ^10^, ^11^ Due to the complex distribution of HCQ, concentration versus time profiles observed after a single HCQ intravenous (i.v.) dose in humans are best fit with a three‐compartment PK model.^4^ The terminal half‐life (*t*_1/2_) is ~30–40 days, and it takes approximately 6 months of dosing for HCQ concentrations to reach a steady state.^4^, ^5^ HCQ and its metabolites distribute extensively into red blood cells and the observed blood to plasma concentration ratio (B/P) demonstrates significant variability.^4^, ^6^ After a single 310 mg i.v. dose, the reported B/P of HCQ ranged from 1–22 with an average of 7.2 from paired plasma and blood concentrations collected over approximately 6 months.^4^ Furthermore, after daily dosing for 6 months, the B/P for HCQ and its primary metabolite desethylhydroxychloroquine (DHCQ) were similar with values ranging from 2.5 to 10.^6^

While it was established more than 30 years ago that ~70% of HCQ is eliminated by metabolism, the cytochrome P450 (CYP) isoenzymes contributing to HCQ elimination in vivo were only recently identified.^12^ HCQ is predominantly eliminated by CYP2C8 with minor contributions from CYP2D6 and CYP3A4,^12^ and all three CYP enzymes form the metabolite DHCQ. DHCQ is further metabolized to desethylchloroquine (DCQ) and bisdesethylhydroxychloroquine (BDCQ). Although contributions of specific CYPs to DHCQ metabolism have not yet been elucidated, it has been established that HCQ, DHCQ, DCQ, and BDCQ are all eliminated in part through renal excretion.^6^ Disease‐induced alterations in CYP‐mediated metabolism and/or renal function may lead to changes in exposure of HCQ and its three metabolites, which may impact efficacy and/or safety outcomes.

In the early stages of the COVID‐19 pandemic, HCQ was investigated as a therapeutic option based on early evidence demonstrating in vitro antiviral activity against SARS‐CoV‐2 and past evidence of antiviral activity against related viruses.^13^, ^14^, ^15^ The established safety profile of HCQ facilitated the rapid implementation of numerous clinical trials, including two clinical trials to determine utility of HCQ as either a post‐exposure prophylaxis (PEP) or early treatment for COVID‐19 where participants in the PEP and treatment trials were SARS‐CoV‐2 negative [(−)] and positive [(+)], respectively (ClinicalTrials.gov; NCT04328961, NCT04354428).^16^, ^17^ Although HCQ was ineffective in both clinical trials, understanding HCQ and metabolite PK is important since it may be repurposed for other disease states. When HCQ dosing regimens were selected for these two studies in the early stages of the COVID‐19 pandemic, it was not yet known that COVID‐19 was associated with changes in the activity of multiple CYPs.^18^, ^19^ However, given the uncertainty about the potential disease impact on HCQ PK, a voluntary PK substudy was incorporated into the clinical trial design of both studies. A physiologically based pharmacokinetic (PBPK) modeling approach incorporating PK data collected from both trials was used to investigate how potential SARS‐CoV‐2 infection/COVID‐19 impacts on HCQ metabolism and could influence HCQ and DHCQ PK. In addition, PBPK modeling was used to further demonstrate the significant contribution of compound blood partitioning to the large degree of observed variability in HCQ and DHCQ PK.

## METHODS

### Participant characteristics

Dried blood spot (DBS) samples were collected from participants who volunteered for PK substudies within two separate clinical trials investigating HCQ as a PEP or treatment of COVID‐19. A total of 407 SARS‐CoV‐2(−) and 148 SARS‐CoV‐2(+) participants received HCQ as part of the assigned clinical trial intervention.^16^, ^17^ Participants who received HCQ in either clinical trial and contributed at least one DBS with quantifiable HCQ and/or DHCQ were included in the present analysis. Of the total study participants administered HCQ, 94/407 SARS‐CoV‐2(−) (*n* = 226 HCQ samples, *n* = 197 DHCQ samples) and 34/148 SARS‐CoV‐2(+) (*n* = 123 HCQ samples, *n* = 115 DHCQ samples) participants volunteered to contribute DBS with complete information to identify dosing and sampling times for the PK analysis. Data from the volunteers was self‐reported as both substudies were conducted remotely. All participants were instructed to provide an accurate record of the time of the dose prior to the dried blood sampling and an accurate time of the blood sampling. Participants in the substudy were requested to provide whole blood one to five times after dosing had commenced. Additional instructions on how to provide the dried blood spots were distributed to study participants upon enrollment in the substudy. The PEP study was conducted in participants identified as a close contact of a person with a polymerase chain reaction (PCR)‐confirmed SARS‐CoV‐2 infection. Participants in the PEP study were administered 400 mg HCQ daily for 3 days followed by 200 mg HCQ daily for 11 days (ClinicalTrials.gov; NCT04328961) or placebo.^16^ Study participants from the PEP study who tested positive before the end of the study were not included in the SARS‐CoV‐2(−) participant group. For HCQ treatment of mild COVID‐19, non‐hospitalized participants with laboratory‐confirmed SARS‐CoV‐2 infection were randomized to receive 400 mg HCQ twice daily for 1 day followed by 200 mg HCQ twice daily for the remaining 9 days with or without azithromycin (ClinicalTrials.gov; NCT04354428) or placebo‐equivalent.^17^ A subset of SARS‐CoV‐2(+) participants providing HCQ and DHCQ PK data (*n* = 12) received concomitant azithromycin for 5 days as part of the clinical trial design.^17^ Azithromycin has been reported as a reversible and time‐dependent CYP3A4 inhibitor in vitro.^20^, ^21^ Clinical drug–drug interaction studies and case reports investigating azithromycin as a potential CYP3A inhibitor have produced conflicting results with CYP3A‐sensitive substrates.^22^, ^23^, ^24^ Since HCQ metabolism through CYP3A is a relatively minor elimination pathway (~15% of total clearance), the potential impact of azithromycin on HCQ exposure is expected to be negligible. Thus, all participants, regardless of those who received azithromycin, were included in the SARS‐CoV‐2(+) cohort. The Western Institutional Review Board approved these studies with reliance agreements with the collaborating institutions. The studies were registered with ClinicalTrials.gov (NCT04328961 and NCT04354428).

### Sample preparation and LC–MS/MS analysis

A Shimadzu Nexera ultra‐performance liquid chromatography (UPLC) system equipped with two pumps (LC‐30AD), column oven (CTO‐30AS), and an auto‐sampler (SIL‐30AC) were used for analyte separation. Mass spectrometric detection was performed utilizing a liquid chromatography with tandem mass spectrometry (LC–MS/MS) 8060 system (Shimadzu Scientific Instruments), equipped with a DUIS source operated in positive electrospray ionization mode. Chromatographic separations were performed with an Aquacil C18 (5 μm, 50 × 4.6 mm; ThermoScientific) and protected with a C18 guard column (Phenomenex). The mobile phase consisted of 0.2% formic acid in water (mobile phase A) and methanol (mobile phase B) at a total flow rate of 0.5 mL/min. The chromatographic separation was achieved using a 10‐min gradient elution. All analytes were simultaneously extracted from DBS to prepared quality control and calibration standards by protein precipitation followed by phospholipid removal utilizing Phree 96 well plates (Phenomenex). A BSD600 Duet device (Model No. BSD600/DUET; BSD Robotics) was used to cut two DBS for all calibration curve (CC), quality controls (QCs), and human DBS samples. Briefly, two 3 mm DBS punches were collected in deep 96‐well plate and diluted with 100 μL 1% formic acid in water and 10 μL mix internal standard solution (0.2 μg/mL). The 96 plates were then vortexed for 5 min and sonicated for 10 min. Ice‐chilled methanol (350 μL) was added to each well of the 96‐well plate and vortexed for 5 min and then transferred to a Phree 96‐well plate and centrifuged at 3000 revolutions/min for 10 min. After centrifugation, 70 μL of the eluate was collected and diluted with 70 μL 0.1% formic acid in water and 10 μL were injected into the LC–MS/MS system. The assay was linear over the concentration range of 1–2000 ng/mL for all analytes, and standard and control concentrations were within 90% of expected values.

### PBPK modeling software

A previously published PBPK model (Simcyp, version 18; Certara USA) was used as the base model for our studies (Table S1, referred to as “base model” throughout the article).^12^ The base model was developed to support HCQ dosing regimens for treatment of COVID‐19. For the current analysis, the PBPK model used to simulate the concentration versus time profiles of HCQ and DHCQ in SARS‐CoV‐2 (−)/(+) populations was developed in Simcyp version 21.

### Blood to plasma concentration ratio (B/P) variability

Simcyp does not provide an option for the user to incorporate a range of B/P values across a study population or to input a value for the coefficient of variation for a compound's B/P. Therefore, to capture the range of B/P values for HCQ and DHCQ expected across a study population, the HCQ and DHCQ blood levels were simulated for each individual dataset for each population. The B/P range of 1–12 includes 90% of observations in a previously reported single dose i.v. study,^4^ and the frequency data in this range were applied to varying population sizes. In addition, this range is similar to what was reported for HCQ and DHCQ (B/P = 2.5–10) after 6 months of daily HCQ administration.^6^ The B/P for DHCQ was set to the same value as HCQ based on similarity in both the reported B/P^6^ and molecular structures (e.g., similar p*K*a and log*P* values). Additional information on incorporating B/P frequency based on the population size is described in Table S1.

### Optimization of HCQ and DHCQ distribution and DHCQ clearance

The tissue–plasma partition coefficient (*K*_p_) values for HCQ and DHCQ were modified to capture the blood concentration versus time profiles observed over 4000 h after a single HCQ i.v. dose.^4^, ^5^ To identify a set of *K*_p_ values that best described the distribution of HCQ and DHCQ, *K*_p_ values for each organ were predicted using Simcyp's Method 2 with B/P values of 1, 7, and 12. Each distribution model was tested using the model evaluation metrics described below. To improve model fit, adipose and muscle *K*_p_ values were adjusted to capture the observed extended terminal elimination phase. Next, the appropriate fit of DHCQ clearance was assessed following the finalization of HCQ and DHCQ distribution. Initial parameter estimation in Simcyp was used to assist in the determination of an appropriate DHCQ clearance by fitting DHCQ concentrations observed after i.v. infusion of HCQ.^4^ A sensitivity analysis was then conducted to determine the best fit of the model to the observed DHCQ concentration points collected from SARS‐CoV‐2(−) study participants.

### Mild COVID‐19 virtual population

The optimized PBPK model was used to investigate how HCQ and DHCQ PK are expected to change in response to cytokine‐induced CYP changes in mild COVID‐19 disease.^25^ The Simcyp healthy volunteer population was used to represent the SARS‐CoV‐2(−) group. A population representing patients with mild COVID‐19 was built by modifying the healthy volunteer population with a 30% reduction in the mean expression of CYP3A4 and a 60% reduction in the mean expression of CYP2C8. The reduction in CYP3A and lack of change in CYP2D6 metabolic activity was demonstrated with a Geneva Cocktail study conducted in hospitalized COVID‐19 patients.^25^ There was no in vivo data to support changes in CYP2C8 activity associated with SARS‐CoV‐2 infection/COVID‐19. However, an in vitro study evaluating the impact of cytokines known to be elevated in COVID‐19 patients (IL‐6, TNF‐α, IL‐1β, and IFN‐λ) observed a 60% reduction in CYP2C8 mRNA in the presence of these cytokines, and this served as a rationale for the 60% reduction in mean expression of CYP2C8 in the COVID‐19 virtual population.^26^, ^27^ Although assumptions were made regarding the extrapolation of CYP mRNA data to enzyme expression, this was taken as a “worst case scenario” approach to reflect the highest possible reduction in CYP2C8 metabolic activity. The changes in mean expression of CYP3A and CYP2C8 was assumed to lead to the same percentage of reduction of clearance through these metabolic pathways. Thus, based on the HCQ fraction metabolized (*f*_m_) and fraction excreted (*f*_e_) values (*f*_m,CYP2C8_ = 0.37, *f*_m,CYP3A_ = 0.17, *f*_m,2D6_ = 0.19, and *f*_e_ = 0.27), the modifications to CYP expression in the mild COVID‐19 population are expected to generate a 28% increase in HCQ exposure compared to the healthy population. To determine the number of participants required to observe a disease effect (i.e., >25% increase in maximum concentration (*C*_max_) and/or exposure) for HCQ and DHCQ, 10 trials of varying population sizes (*n* = 10, 30, 100, 250, and 500) were simulated in a 1:1 ratio of healthy to mild COVID‐19. To evaluate the predicted difference in HCQ and DHCQ PK between the two populations, ratios between the mild COVID‐19 and healthy populations area under the curve (AUCR) and *C*_max_ (*C*_max_R) were evaluated.

### Model evaluation

Models were evaluated by the proportion of observed concentrations falling within the 95% prediction intervals (Equation 1), as well as the average fold error (AFE) and absolute average fold error (AAFE) values (Equations 2 and 3). The prediction intervals were calculated by determining the 5th and 95th percentiles of each analyzed dataset where *n* = ordinal rank of the value within the dataset, P = percentile, and *N* = number of values within the dataset. Optimal model performance was aimed to maximize the proportion of observed concentrations falling within the 95% prediction intervals (abbreviated as WPI) and to achieve AFE and AAFE values within a range of 0.33–3.0.

(1) $$n=\left(\frac{P}{100}\right)x\;N$$

(2) $$\mathrm{AFE}=10^{\frac{1}{N}\sum \left({\mathrm{Log}}_{10}\frac{\text{Predicted}}{\text{Observed}}\right)}$$

(3) $$\text{AAFE}=10^{\frac{1}{N}\sum \left(|{\mathrm{Log}}_{10}\frac{\text{Predicted}}{\text{Observed}}|\right)}$$

### Model validation

Previously published PK datasets for HCQ and its metabolite DHCQ were used to assess model performance beyond the SARS‐CoV‐2(−)/(+) populations. This analysis incorporated nine of the studies used to validate the base PBPK model (Table S3).^12^ Two studies used to validate the base model were excluded from this analysis because the datasets were used to optimize HCQ and DHCQ distribution and DHCQ clearance for this final proposed PBPK model. Data were extracted from plots in each publication using GraphGrabber (v2.0.2; Quintessa). Ten trials of 30 subjects (*n* = 300 total subjects) from the Simcyp default healthy Caucasian population were simulated for each dataset. The model was run in the same manner described in the model building process which included incorporating B/P variability and optimized HCQ and DHCQ distribution and DHCQ clearance values. Evaluation of model performance included assessing the proportion of observed datapoints within the prediction interval and calculating AFE and AAFE using Equations 2 and 3. Acceptable AFE and AAFE values were between 0.33 and 3.0.

### Statistical testing

A statistical analysis was conducted to determine significant differences between study participant demographics and disease‐mediated changes in HCQ and DHCQ AUC and *C*_max_ values. Unpaired Student's *t*‐tests were conducted with the arithmetic means of healthy and mild COVID‐19 populations of sizes *n* = 10, 30, 100, 250, and 500 since the distribution of values were assumed to follow a normal distribution. A *p* value ≤0.05 was considered statistically significant.

## RESULTS

**FIGURE 1:**
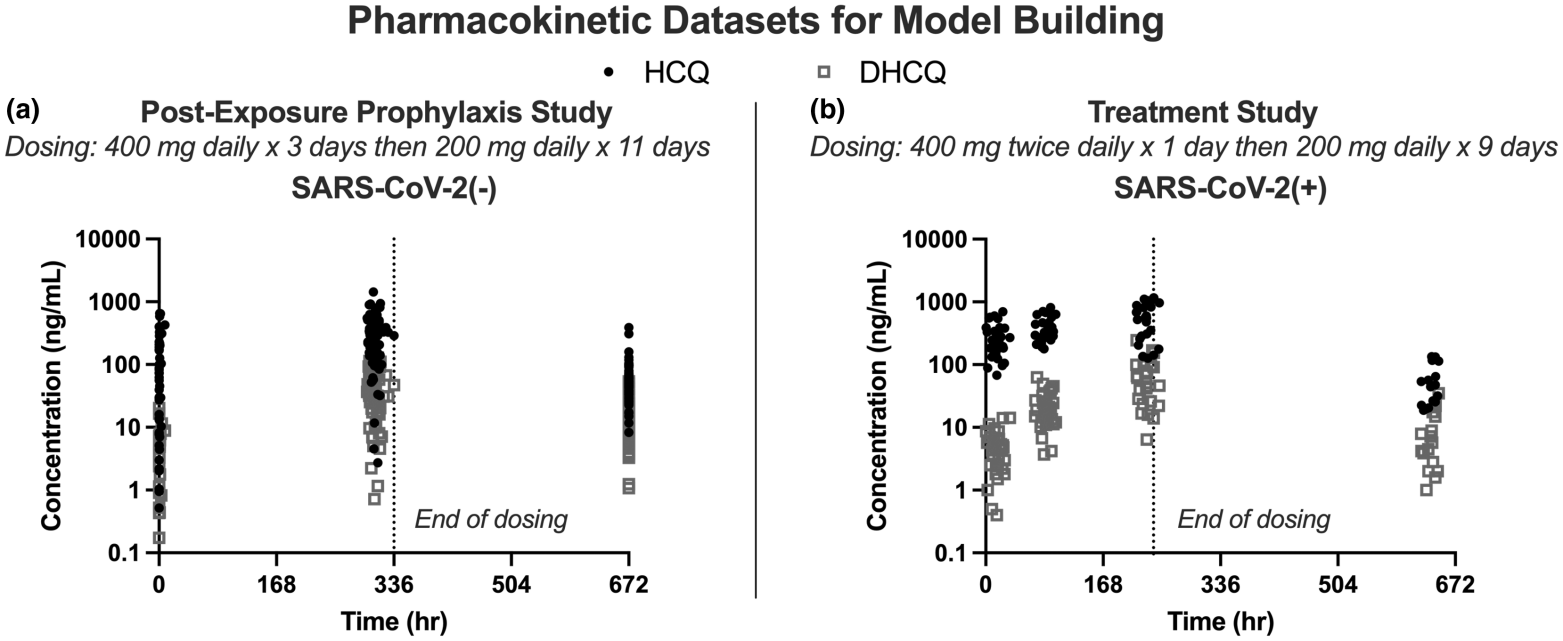
Pharmacokinetic data for hydroxychloroquine (HCQ) and desethylhydroxychloroquine (DHCQ) in SARS‐CoV‐2 (−)/(+) participants. HCQ and DHCQ concentrations were determined for 94 SARS‐CoV‐2(−) participants who received HCQ 200 mg twice daily for 3 days followed by 200 mg daily for 11 days (a). HCQ and DHCQ concentrations were determined for 34 SARS‐CoV‐2(+) participants who received HCQ 400 mg twice daily for 1 day followed by 200 mg twice daily for 9 days (b). Significant variability in HCQ and DHCQ concentrations was observed within both study groups.

**FIGURE 2:**
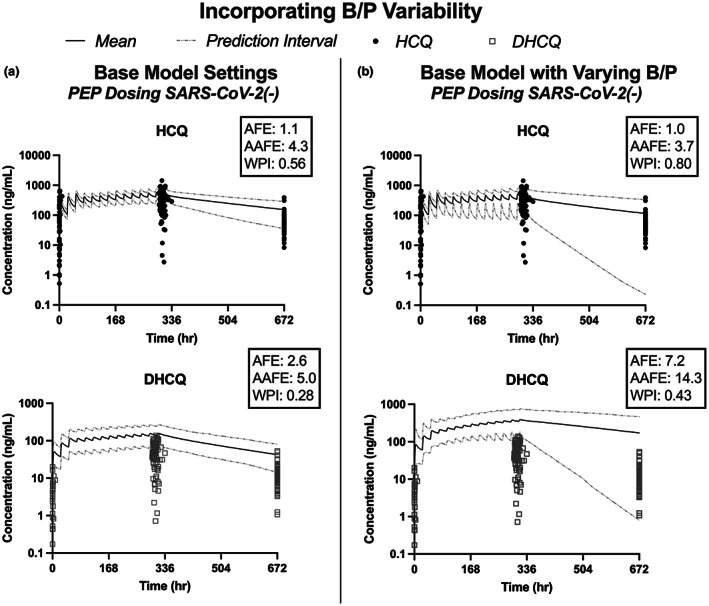
Base physiologically based pharmacokinetic (PBPK) model performance with hydroxychloroquine (HCQ) and desethylhydroxychloroquine (DHCQ) pharmacokinetic (PK) data observed in SARS‐CoV‐2(−) participants. The base model does not account for interindividual variability in HCQ and DHCQ concentrations (a). The PBPK model performance was improved by incorporating the known range of blood to plasma concentration ratios for each compound (b). AAFE, absolute average fold error; AFE, average fold error; B/P, blood to plasma concentration ratio; DHCQ, desethylhydroxychloroquine; HCQ, hydroxychloroquine; PEP, post‐exposure prophylaxis; WPI, proportion within 95% prediction intervals.

A total of 128 participants [*n* = 34 SARS‐CoV‐2(+) and *n* = 94 SARS‐CoV‐2(−)] contributed at least one DBS with quantifiable HCQ and/or DHCQ (Figure 1). No significant difference in demographic data was observed between SARS‐CoV‐2(−)/(+) participants (Table 1). When a previously published PBPK model was used to fit the HCQ and DHCQ data observed in the SARS‐CoV‐2(−) cohort, the model did not adequately capture HCQ and DHCQ variability indicated by the large proportion of concentrations observed outside the 95% prediction interval for both HCQ and DHCQ. In addition, the model overestimated DHCQ concentrations (Figure 2a). To improve the model fit, a distribution of previously reported B/P values ranging from 1 to 12 for both HCQ and DHCQ was incorporated into the model, and the proportion of observed values within the prediction interval for HCQ improved from 0.56 to 0.80 (Figure 2). However, when incorporating the B/P distribution for DHCQ, the model overpredicted DHCQ concentrations (AFE = 7.2), suggesting further optimization of distribution and/or clearance were necessary to improve model fit (Figure 2b).

**TABLE 1:** Demographic data for pharmacokinetic substudy participants.

	SARS‐CoV‐2(−): PEP (*n* = 94)					SARS‐CoV‐2(+): Treatment (*n* = 34)				
	*NCT04328961*					*NCT04354428*				
	Asian	Black or African American	White	Other	Native American or Alaskan Native	Asian	Black or African American	White	Other	*P* value
Ethnicity (*N* (%))	7 (7)	5 (5)	72 (77)	10 (11)	8 (24)	2 (6)	2 (6)	21 (62)	1 (2)	0.14
	**Min**	**Max**	**Median**		**Min**	**Max**	**Median**			
Age (years)	18	76	39		21	71	37			0.91
	**Min**	**Max**	**Median**		**Min**	**Max**	**Median**			
Weight (lb)	110	430	173		115	272	171			0.89
	**Female**	**Male**			**Female**	**Male**				
Sex at birth (*N* (%))	56 (60)	38 (40)			25 (74)	9 (26)				0.15

*Note*: All participants included in this analysis contributed at least one dried blood spot sample. An unpaired Student's *t*‐test was conducted to identify significant differences between study participant demographics and no significant difference was observed between the two groups (*p* value > 0.05).

Abbreviation: PEP, post‐exposure prophylaxis.

**FIGURE 3:**
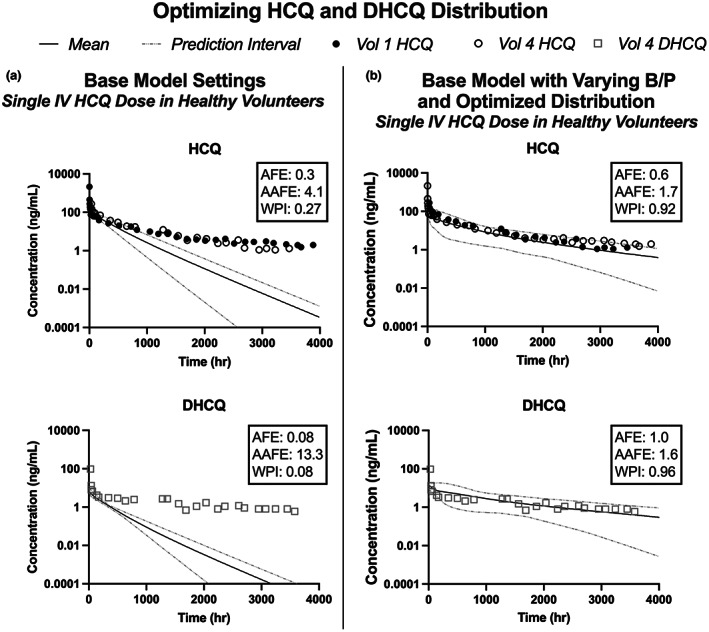
Hydroxychloroquine (HCQ) and desethylhydroxychloroquine (DHCQ) distribution parameters within the physiologically based pharmacokinetic (PBPK) model were optimized using pharmacokinetic (PK) data collected over 4000 h after a single intravenous dose of HCQ to two volunteers. The observed data were extracted from the two studies.^4^, ^5^ The base model does not capture the terminal phase of HCQ and DHCQ concentrations (a). After incorporating the blood to plasma concentration ratio variability and optimizing tissue–plasma partition coefficients (*K*_p_) for HCQ and DHCQ, the model fit improves for both HCQ and DHCQ (b). AAFE, absolute average fold error; AFE, average fold error; B/P, blood to plasma concentration ratio; DHCQ, desethylhydroxychloroquine; HCQ, hydroxychloroquine; Vol 1, Volunteer 1; Vol 4, Volunteer 4; WPI, proportion within 95% prediction intervals.

HCQ is known to have a large and variable *V*_D_. Thus, parameters within the PBPK model influencing the distribution of each compound (e.g., organ *K*_p_ values) were investigated to determine how the changes in *K*_p_ values impacted model fit. Previously published HCQ and DHCQ concentration versus time profiles observed after a single i.v. infusion of 310 mg HCQ were used to optimize HCQ and DHCQ distribution (Figure 3a).^4^, ^5^ The previously published base PBPK model underpredicted HCQ and DHCQ concentrations for the terminal portion of the concentration versus time profile. To improve the model fit, the PBPK model was modified by assessing *K*_p_ values that best described the extended terminal elimination phase while also optimizing the *K*_p_ scalar for both HCQ and DHCQ. The base PBPK model settings applied a *K*_p_ scalar of 2.2 only to HCQ, but not to DHCQ (base model DHCQ *K*_p_ scalar = 1). For the current model, the *K*_p_ scalar of 2.2 was also applied to DHCQ based on the assumption that both compounds would have comparable distribution based on the similarity between molecular structures (e.g., conserved ionizable groups and similar molecular weights/logP). The optimized distribution was tested by simulating a population of 30 participants receiving a 310 mg single dose i.v. infusion of HCQ and comparing the simulated values to the observed individual concentration profiles (Figure 3b). The AUC ratio of the predicted to observed data for both HCQ and DHCQ was within two‐fold, and the 95% prediction intervals captured >90% of the observed datapoints indicating acceptable model performance. While the AFE were low (HCQ AFE = 0.3, DHCQ AFE = 0.08) and AAFE were high (HCQ AAFE = 4.1, DHCQ AAFE = 13.3) with the base model (Figure 3a), the AFE and AAFE for HCQ and DHCQ were between 0.6 and 1.7 with the updated distribution settings (Figure 3b).

**FIGURE 4:**
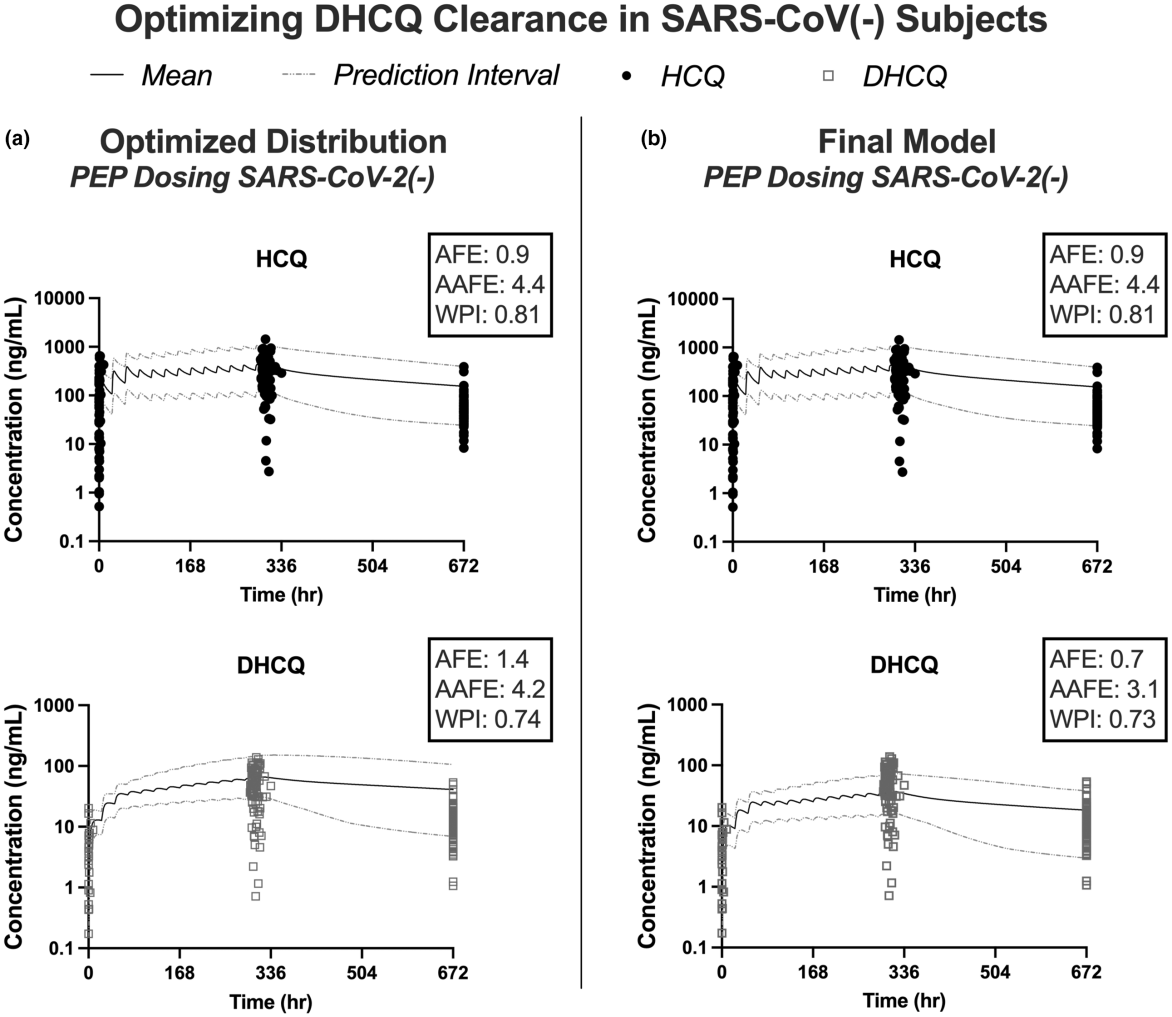
Desethylhydroxychloroquine (DHCQ) clearance was optimized using the DHCQ concentrations observed in SARS‐CoV‐2(−) participants. Using the updated distribution model, the model overestimates DHCQ exposure (a). As expected, the optimized clearance value for DHCQ improves the model fit (b). AAFE, absolute average fold error; AFE, average fold error; DHCQ, desethylhydroxychloroquine; HCQ, hydroxychloroquine; PEP, post‐exposure prophylaxis; WPI, proportion within 95% prediction intervals.

After optimizing the distribution parameters and incorporating the range of B/P values, the updated model was used to simulate HCQ and DHCQ concentrations in 10 trials of 30 participants, and these values were compared to the SARS‐CoV‐2(−) dataset (Figure 4a). While the proportion of datapoints within the 95% prediction intervals increased compared to the proportion captured in the prediction intervals in the base model, DHCQ concentrations were still overpredicted. For DHCQ elimination, the base model incorporated the observed renal clearance (Cl_R_ = 2.9 L/h),^6^ the Simcyp predicted B/P value of 1.7, and enzyme kinetic data to fit the remaining metabolite clearance to previously published DHCQ data. For the current model, the DHCQ concentrations observed with a single HCQ i.v. infusion dose were used to optimize the whole organ metabolic clearance using parameter estimation within Simcyp.^4^ While the final DHCQ AAFE was slightly outside of the desired range (AAFE = 3.1), the AFE and AAFE were improved compared to the base model (Figure 4b vs. Figure 2b).

**FIGURE 5:**
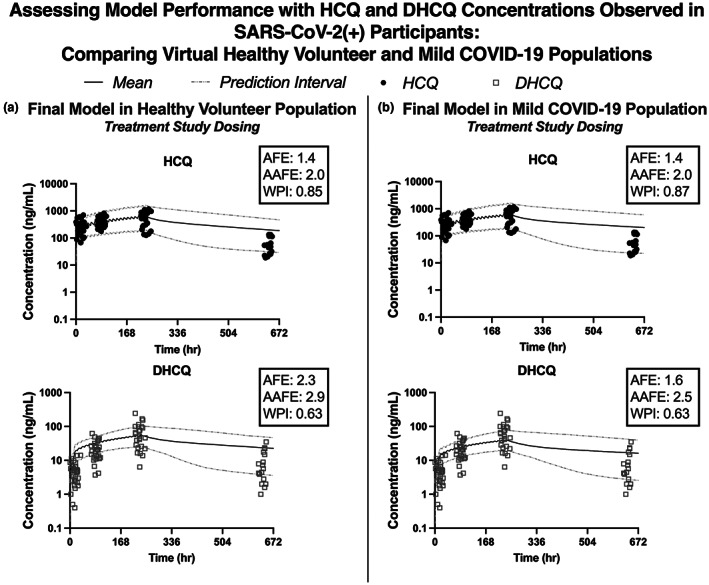
Model performance in SARS‐CoV‐2(+) participants. The optimized physiologically based pharmacokinetic (PBPK) model incorporated the dosing regimen for SARS‐CoV‐2(+) participants (400 mg twice daily x 1 day followed by 200 mg twice daily x 9 days) in a virtual population of healthy volunteers (a) and mild SARS‐CoV‐2(+) participants (b). Overall, the model captures the observed dataset prior to incorporating changes in cytochrome P450 activity that may be observed with mild COVID‐19 disease. AAFE, absolute average fold error; AFE, average fold error; DHCQ, desethylhydroxychloroquine; HCQ, hydroxychloroquine; WPI, proportion within 95% prediction intervals.

The final optimized model was fit to the HCQ and DHCQ concentrations versus time data observed in SARS‐CoV‐2(+) participants to determine if HCQ and DHCQ exposure was altered by SARS‐CoV‐2(+) infection/presence of COVID‐19 disease manifestations (Figure 5). The Simcyp default healthy population was used for the initial simulations with 10 trials of 30 participants and model performance was adequate for HCQ and DHCQ in both healthy and mild SARS‐CoV‐2 infection since the AFE and AAFE values for both compounds were between 1.4 and 2.9 (Figure 5a,b). Previous studies have established that CYP function may change as consequence of COVID‐19.^25^, ^26^, ^27^ It is likely that our dataset was not able to detect a COVID‐19 impact on HCQ PK because the anticipated change in HCQ AUC is small (<30% increase) and there is a large degree of variability in HCQ and DHCQ exposure.

To determine how many participants would be required to detect a significant increase in HCQ AUC due to COVID‐19‐induced changes in CYP metabolism, a mild COVID‐19 virtual population was generated to reflect the previously reported changes in CYP function.^25^, ^27^ The HCQ PBPK model was used to simulate HCQ and DHCQ concentrations in healthy and mild COVID‐19 populations, and the significance of the change in HCQ and DHCQ *C*_max_ and AUC_0–672h_ was assessed to demonstrate the total predicted effect of a potential COVID‐19‐induced reduction in CYP activity. For the healthy and COVID‐19 populations, 10 trials were simulated with *n* = 10, 30, 100, 250, and 500 participants in each trial. The dosing regimen administered in the HCQ treatment study (ClinicalTrials.gov; NCT04354428) was used for the simulations.^17^ The PK parameters and ratios for simulated healthy and COVID‐19 participants are summarized in Table 2 for trials with *n* = 300 and 5000 participants in each group. The remaining populations (*N* = 100, 1000, and 2500) are summarized in Table S2. There was minimal difference in model performance when using the Simcyp healthy volunteer population in comparison to the mild COVID‐19 population that incorporated predicted CYP expression changes due to COVID‐19 as demonstrated by the AFE and AAFE values (Figure 5). A population size of 5000 in both arms was necessary to identify a statistically significant difference in the HCQ AUC_0–672h_ and *C*_max_ (*p* < 0.05), but fewer (1000 in each arm) was necessary to identify a statistically significant difference in the DHCQ AUC_0–672h_ and *C*_max_. In addition, the simulation results suggest that >5000 participants in each arm would be required to detect a >25% increase in HCQ AUC_0–672h_ in subjects with mild COVID‐19 compared to healthy participants.

**TABLE 2:** Simulated area under the curve and maximum concentration values for hydroxychloroquine and desethylhydroxychloroquine in healthy and mild COVID‐19 populations.

			Healthy		Mild COVID‐19					
			HCQ	DHCQ	HCQ	DHCQ	Ratio (mild COVID‐19/healthy)			
	
Sample size for each population	Parameter	Units	Mean (SD)	Mean (SD)	Mean (SD)	Mean (SD)	HCQ	*P* value	DHCQ	*P* value
*N* = 300: 10 trials of 30 participants in each population	*C*_max_	ng/mL	516 (326)	77 (51)	517 (290)	57 (34)	1.0	0.99	0.74	0.08
	AUC_0–672_	ng h/mL	173,388 (123,128)	29,872 (21,989)	176,031 (114,765)	22,122 (15,591)	1.0	0.93	0.74	0.12
*N* = 1000: 10 trials of 100 participants in each population	*C*_max_	ng/mL	504 (294)	79 (52)	534 (289)	61 (42)	1.1	0.47	0.77	0.01**
	AUC_0–672_	ng h/mL	167,115 (111,851)	30,510 (22,302)	182,745 (116,700)	24,136 (19,484)	1.1	0.33	0.79	0.03*
*N* = 5000: 10 trials of 500 participants in each population	*C*_max_	ng/mL	492 (270)	77 (48)	531 (277)	57 (37)	1.1	0.02*	0.74	<0.001***
	AUC_0–672_	ng h/mL	162,982 (102,687)	29,494 (21,251)	182,044 (111,827)	22,452 (16,911)	1.1	0.005**	0.76	<0.001***

*Note*: HCQ and DHCQ AUC_0–672h_ and *C*_max_ values in healthy and mild COVID‐19 populations were calculated using concentration versus time profiles simulated in 10 trials of *n* = 30, 100, and 500 participants. Ratios of the HCQ and DHCQ AUC_0‐672h_ and *C*_max_ values were determined (mild COVID‐19/healthy), and an unpaired Student's *t*‐test (*α* = 0.05) was conducted to identify significant differences in HCQ and DHCQ AUC_0–672h_ and *C*_max_ values when comparing healthy and mild COVID‐19 populations. As shown in the table, a total of 1000 participants in both study arms would be necessary to see a statistically significant change in DHCQ pharmacokinetic parameters, but more are required (*n* = 5000) to observe a significant increase in HCQ AUC_0–672h_ and *C*_max_.

Abbreviations: AUC_0–672h_, area under the curve from 0 to 672 h; *C*_max_, maximum concentration; DHCQ, desethylhydroxychloroquine; HCQ, hydroxychloroquine; SD, standard deviation.

Significance denoted with asterisks: *p≤0.05, **p≤0.01, ***p≤0.001.

Finally, model performance in simulating previously published PK datasets for HCQ and DHCQ following HCQ administration was conducted to test the robustness of the current model. A summary of each study and associated model performance measures are summarized Table S3 and Figures [Link], [Link].^6^, ^28^, ^29^, ^30^, ^31^, ^32^, ^33^, ^34^, ^35^ The proposed model performed well with a majority of the test datasets for both HCQ plasma and blood concentrations. These test datasets included oral and intravenous administration in addition to healthy volunteers and patients with lupus and rheumatoid arthritis which suggests route of administration and disease effects have minimal impact on the PBPK model performance. Additionally, since blood concentrations were used in our model development process, this analysis supports the use of the current model for HCQ plasma simulations also. When validating the model performance for DHCQ, concentrations were underpredicted for two of three studies that measured DHCQ levels (Table S3).

## DISCUSSION

The optimized HCQ and DHCQ PBPK model incorporating B/P variability provides a valuable tool that can be used to support the design of future clinical trials with HCQ. While a previously published PBPK model provided a foundation for this analysis, it did not capture the observed variability in HCQ and DHCQ concentrations observed in both the SARS‐CoV‐2(−)/(+) populations. The improvement in model performance gained by incorporating a range of B/P for HCQ and DHCQ demonstrates how this same technique could be used for other compounds that have high B/P with similar degrees of observed PK variability.

The initial goals of this study were to determine whether there was a relationship between HCQ concentrations and SARS‐CoV‐2 viral clearance and to identify potential SARS‐CoV‐2 infection‐associated changes in HCQ PK. While it has been repeatedly established that HCQ provides no benefit for treatment of COVID‐19,^3^, ^17^, ^36^, ^37^, ^38^ it remained unclear whether HCQ exposure in those with SARS‐CoV‐2 infection was different than in healthy participants. Our studies demonstrated that a previously published HCQ and DHCQ PBPK model did not adequately fit HCQ and DHCQ PK data observed in our SARS‐CoV‐2(−)/(+) populations. Specifically, the variance of HCQ and DHCQ concentrations was not captured by the base model and DHCQ concentrations were overpredicted. The variance of HCQ and DHCQ concentrations was adequately captured by our modified PBPK model after incorporating the known variability in B/P, further supporting the considerable role of blood partitioning in the variability observed in both HCQ and DHCQ exposure.

Incorporating modifications to HCQ and DHCQ distribution in the PBPK model notably improved the fit for HCQ and DHCQ concentrations observed after a single i.v. dose PK study that extensively collected blood samples over 4000 h.^4^, ^5^ Specifically, optimizing the organ *K*_p_ values for HCQ and DHCQ greatly improved the predicted distribution behavior and aided in capturing the terminal elimination phase. Given the relatively short HCQ dosing regimens for COVID‐19 treatment/prophylaxis and sparse blood sampling, the changes to HCQ and DHCQ distribution had negligible impact on the model performance with the clinical study PK data reported in this article. However, the optimized distribution settings will improve confidence in model application for studies with prolonged dosing/sampling regimens.

After incorporating B/P variability and optimizing HCQ and DHCQ distribution, DHCQ concentrations were still overpredicted and DHCQ clearance required further optimization. After increasing the metabolic clearance for DHCQ, the model adequately described the SARS‐CoV‐2(−) HCQ and DHCQ datasets. It should be noted that the base model fit DHCQ clearance using in vivo data and the Simcyp predicted B/P = 1.7, and the clearance of the optimized model needed to be adjusted to account for the change in the mean B/P. The same change was not necessary for HCQ as the mean B/P was similar to the base model when incorporating the frequency distribution of B/P values. Although multiple changes were made to the original model, the final model performance proved to be robust for HCQ, supporting application for predictions of HCQ concentrations in other scenarios. In contrast, model performance for DHCQ was inconsistent with external datasets as DHCQ concentrations were underpredicted in some studies after multiple dose administration of HCQ. The cause of the inconsistency is unclear and will require further study.

To demonstrate how the optimized HCQ PBPK model could be applied to clinical trial design, simulations of HCQ administration to healthy and mild COVID‐19 populations were performed to identify how many participants would be necessary to detect a significant change in HCQ and DHCQ PK. The predicted COVID‐19‐induced reduction in the mean expression of CYP3A4 (30%) and CYP2C8 (60%) was based on previously published data.^25^, ^27^ The changes in CYP function were incorporated into a virtual mild COVID‐19 population, and it was observed that it would take more than 5000 volunteers in both the healthy and COVID‐19 cohorts to detect a >25% increase in the HCQ AUC, although a statistically‐significant change in the HCQ AUC and *C*_max_ could be observed with 10 trials of *n* = 500 participants (5000 total in each arm). Alternatively, it would take 10 trials of *n* = 100 participants (*n* = 1000 total in each arm) to see a statistically significant change in DHCQ AUC and *C*_max_. Overall, this exercise demonstrates that incorporating B/P variability may impact study participant recruitment as a large number of participants would be required to detect disease‐associated changes in HCQ and DHCQ *C*_max_ and/or AUC.

Although model performance was significantly improved by the changes described in this article, limitations were present in this analysis. The remaining variability observed in this dataset that is not captured by the model could be attributed, in part, to the fact that sampling and dosing data were self‐reported by the participants assessed in this clinical trial. Both clinical trial datasets included remote reporting and handling of DBS and were completely reliant on accurate reporting by study participants. Variability could also be attributed to study participant adherence which is also reliant on accurate study participant reporting of dose intake. Another limitation of this model is based on the optimized DHCQ metabolic clearance as it appears it may limit extrapolation of this model beyond short‐term dosing. Lastly, the predicted change in mean expression for CYP2C8 was based on in vitro mRNA changes in the presence of cytokines and may not reflect the true in vivo change in CYP2C8 activity. Similarly, the predicted change in CYP3A was based on hospitalized patients with COVID‐19 who would most likely have a more severe disease presentation than the population included in this analysis. Furthermore, the model assumes instantaneous distribution from plasma to blood which may not reflect HCQ and DHCQ partitioning in vivo.

In conclusion, while it is clear HCQ did not demonstrate benefit over standard of care for COVID‐19, our linked HCQ and DHCQ PBPK model developed with PK data from COVID‐19 trials provides valuable information for HCQ's current and future use across a broad range of indications.^3^ The same techniques will inform the application of PK models to other compounds with extensive, variable blood distribution.

## AUTHOR CONTRIBUTIONS

C.S., Y.S.C, J.S., H.L., K.B.H., M.L.K., M.P.‐O., A.B., H.C.S.K., V.A., M.L., R.L., M.W., D.J.M., C.J., R.V.B., and S.L.M.A. wrote the manuscript and designed the research. C.S., Y.S.C, D.J.M, and S.L.M.A. performed the research. C.S., Y.S.C, D.J.M, and S.L.M.A. analyzed the data.

## FUNDING INFORMATION

C.S. was supported by a National Institute of General Medical Sciences (NIGMS) grant T32 GM007750. R.V.B. reports research support from the Bill and Melinda Gates Foundation (INV‐016204) and the National Institutes of Health. C.J. reports research support from the Bill and Melinda Gates Foundation (INV‐017062 and INV‐01620). D.J.M. was awarded funding from the University of Washington (UWSC11894 and UWSC12049).

## CONFLICT OF INTEREST STATEMENT

A.B. reports research support from the US National Institutes of Health, the Bill and Melinda Gates Foundation, the Foundation for Innovative New Diagnostics, and the New York City Department of Health and Mental Hygiene, and compensation for consulting services from Gates Ventures. C.J. reports consultancies to AbbVie, GSK, and Gilead, and receives research support from Gilead and royalties from UpToDate. R.V.B. reports Regeneron Pharmaceuticals provided conference abstract and manuscript writing support outside the submitted work. R.L. serves on the Scientific Advisory Board for Gilead and Merck in addition to reporting consultancy agreements for Cepheid and Janssen. All the other authors declared no competing interests for this work.

## Supporting information


Table S1

Table S2

Figure S1

Figure S2

Figure S3

Figure S4

Figure S5

Figure S6

Figure S7

Figure S8

Figure S9

Table S3
