# Supplementary material for: A linked physiologically based pharmacokinetic model for hydroxychloroquine and metabolite desethylhydroxychloroquine in SARS‐CoV‐2(−)/(+) populations
Source: Clin Transl Sci. 2023 Apr 29;16(7):1243–57. doi: 10.1111/cts.13527 (PMC10339702; doi:10.1111/cts.13527)
Supplement: Supplementary file 1 — Table S1 [file CTS-16-1243-s012.pdf]

**Supplementary Table 1.** Model parameters from the base model published originally by Zhang, et al. Table is modified from the published base model supplementary material. Parameters that were changed in the optimized model are indicated by an asterisk (*) and the previous input for the updated parameter is indicated by the value included in brackets.	
**Parameter**<br>***updated in model optimization**	**Input Value**<br>***[model parameter from Zhang et al]***
**Hydroxychloroquine (HCQ)**	
Physicochemical properties	
Molecular weight (g/mol)	335.87
Log*P*	3.84
Compound type	Diprotic base
pKa	9.67, 8.27
**Blood to Plasma Partitioning Ratio (B/P)***	**1-12 [7.2][1]**
Hematocrit	45
Plasma f_u	0.48
Absorption	
Absorption model	ADAM
f_a	0.896
k_a (1/h)	0.798
f_u (Gut)	1
Permeability Assay	Caco-2
Apical pH:Basolateral pH	7.4:7.4
Activity	
Caco-2 (10-6cm/s) (HCQ)	3.68
Caco-2 (10-6cm/s) (Cimetidine)	0.92
Caco-2 (10-6cm/s) (Metoprolol)	23.51
DLM Model Options	Particle Population Balance Model
Solid State Specific Parameters	Solid state 1
Particle size distribution	
Monodispersed Radius (μm)	10
Density (g/mL)	1.2
Particle population balance model options	
Number of Particle SizeBins (Simulation)	30
Radius Bounds (Simulation) (μm)	
Minimum	0.1
Maximum	11
Step Type	Uniform step-size
Aqueous Phase Solubility	
Intrinsic Solubility (So)(mg/mL)	0.034
Distribution	

Distribution model	Full PBPK model
V_{ss} (L/kg)*	**673 [255.1]**
Prediction Method	Method 2
Tissue: Plasma Partition Coefficients	
Adipose*	**2,000 [75.74]**
Bone*	**184 [128]**
Brain*	**113 [79.49]**
Gut*	**770 [535.07]**
Heart*	**832 [577.98]**
Kidney*	**673 [467.85]**
Liver*	**1379 [957.03]**
Muscle*	**100 [470.57]**
Skin*	**358 [248.55]**
Spleen*	**763 [530.05]**
Pancreas*	**455 [317.11]**
K_p scalar	**2.2**
Elimination	
Clearance type	Enzyme kinetics
Intrinsic clearance of CYP 2C8 (µL/min/pmol)	0.089
Fraction of unbound drug in the in vitro microsomal incubation	0.5
rCYP system	E.Coli
ISEF	3.63
Intrinsic clearance of CYP 2D6 (µL/min/pmol)	0.211
fu mic	0.5
rCYP system	E.Coli
ISEF	2.49
Intrinsic clearance of CYP 3A4 (µL/min/pmol)	0.0197
fu mic	0.5
rCYP system	E.Coli
ISEF	1.14
Observed renal clearance (L/h)	12.7
Transporter (Permeability Ltd. Organs)	
Caco-2(10-6cm/s) (HCQ)	3.68
F_u mass	0.001
Basolateral Uptake Clint,T	0.2
Basolateral Efflux Clint,T	0.5
**Desethylhydroxychloroquine (DHCQ)**	
Physicochemical properties	
Molecular weight (g/mol)	307.825

Log *P*	3.432
Compound type	Diprotic Base
pKa1	9.45
pKa2	7.15
Blood to Plasma Partitioning Ratio (B/P)*	**1-12 [1.715]**[1]
Hematocrit	45
Fraction unbound in plasma	0.51
Distribution	
Distribution model	Full PBPK model
V_{ss} (L/kg)*	**954 [14.13]**
Prediction Method	Method 2
Tissue:Plasma Partition Coefficients	
Adipose*	**3,000 [4.68]**
Bone*	**183 [7.8]**
Brain*	**111 [5.7]**
Gut*	**765 [28.8]**
Heart*	**826 [30.7]**
Kidney*	**668 [25.2]**
Liver*	**1369 [50.6]**
Muscle*	**100 [25.5]**
Skin*	**356 [13.5]**
Spleen*	**757 [28.5]**
Pancreas*	**452 [17.8]**
Lung*	**137 [5.9]**
K_p scalar*	**2.2 [1]**
Elimination	
Clearance type*	**Whole organ metabolic clearance [enzyme kinetics]**
HLM (µL/min/mg protein)*	**116**
Observed renal clearance (L/h)	2.862

[1]B/P was input as the same value for HCQ and DHCQ based on the frequencies reported in Tett, et al, *Br J Clin Pharmacol*, 1988. The number of subjects corresponding to each B/P value were based on the total population size as shown in the table below. Each row was simulated individually and combined to create the total population dataset.

**B/P Frequency Based on Population Size**					
**B/P Input**	**N per n=10**	**N per n=30**	**N per n=100**	**N per n=250**	**N per n=500**
**1**	0	1	4	10	20
**2**	0	4	12	30	60
**3**	0	3	10	25	50

**4**	0	3	12	30	60
**5**	1	3	8	20	40
**6**	2	3	10	25	50
**7**	4	4	10	25	50
**8**	2	2	8	20	40
**9**	1	3	10	25	50
**10**	0	1	4	10	20
**11**	0	2	8	20	40
**12**	0	1	4	10	20
**Total**	**10**	**30**	**100**	**250**	**500**
