# Supplementary material for: A linked physiologically based pharmacokinetic model for hydroxychloroquine and metabolite desethylhydroxychloroquine in SARS‐CoV‐2(−)/(+) populations
Source: Clin Transl Sci. 2023 Apr 29;16(7):1243–57. doi: 10.1111/cts.13527 (PMC10339702; doi:10.1111/cts.13527)
Supplement: Supplementary file 2 — Table S2 [file CTS-16-1243-s010.pdf]

Table S2. The final PBPK model was used to compare HCQ and DHCQ C_{max} and AUC values predicted in healthy and mild COVID-19 populations. For each population, 10 trial simulations were performed with varying population sizes (n=10, 30, 100, 250, and 500). Ratios of the AUC and C_{max} of varying population sizes are shown. A significant difference was only observed in the n=5,000 population for HCQ parameters, but fewer (n=1,000) were required to see a difference in DHCQ parameters. An unpaired Student's t-test was used to determine significance with a threshold of $P \leq 0.05$. Significance denoted with asterisks (* ≤ 0.05, ** ≤ 0.01, *** ≤ 0.001)

		Healthy				Mild COVID-19							
		HCQ		DHCQ		HCQ		DHCQ		Parameter Ratio (Mild COVID-19/Healthy)			
Sample Size for Each Population	Parameter	Mean	SD	Mean	SD	Mean	SD	Mean	SD	HCQ	P value	DHCQ	P value
N=100: 10 trials of 10 participants in each population	AUC$_{0-672}$ (ng*h/mL)	189865	93691	32860	17271	189001	83927	24227	12831	1.00	0.98	0.74	0.22
	C_{max} (ng/mL)	776	246	83	43	935	210	62	30	1.20	0.14	0.74	0.22
N=300 10 trials of 30 participants in each population	AUC$_{0-672}$ (ng*h/mL)	173388	123128	29872	21989	176031	114765	22122	15591	1.02	0.93	0.74	0.12
	C_{max} (ng/mL)	516	326	77	51	517	290	57	34	1.00	0.99	0.74	0.08
N=1,000: 10 trials of 100 participants in each population	AUC$_{0-672}$ (ng*h/mL)	167115	111851	30510	22302	182745	116700	24136	19484	1.09	0.33	0.79	0.03*
	C_{max} (ng/mL)	504	294	79	52	534	289	61	42	1.06	0.47	0.77	0.01*
N=2,500: 10 trials of 250 participants in each population	AUC$_{0-672}$ (ng*h/mL)	163822	104226	29977	21597	181264	112039	23187	18467	1.11	0.07	0.77	<0.001***
	C_{max} (ng/mL)	495	274	78	50	530	278	59	40	1.07	0.16	0.76	<0.001***
N=5,000: 10 trials of 500 participants in each population	AUC$_{0-672}$ (ng*h/mL)	162982	102687	29494	21251	182044	111827	22452	16911	1.12	0.005**	0.76	<0.001***
	C_{max} (ng/mL)	492	270	77	48	531	277	57	37	1.08	0.02*	0.74	<0.001***
