# Supplementary material for: A linked physiologically based pharmacokinetic model for hydroxychloroquine and metabolite desethylhydroxychloroquine in SARS‐CoV‐2(−)/(+) populations
Source: Clin Transl Sci. 2023 Apr 29;16(7):1243–57. doi: 10.1111/cts.13527 (PMC10339702; doi:10.1111/cts.13527)
Supplement: Supplementary file 3 — Figure S1 [file CTS-16-1243-s011.pdf]

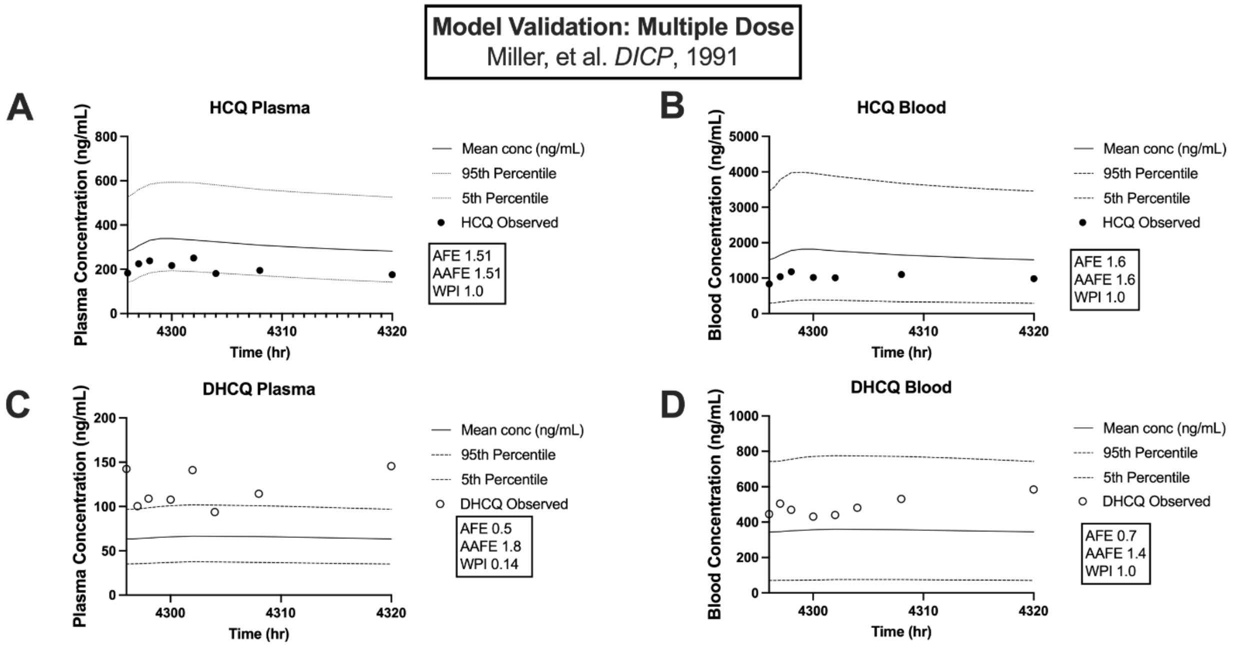

Figure S1: Mean observed (circles) and simulated (solid line) plasma and blood hydroxychloroquine (HCQ) and desethylhydroxychloroquine (DHCQ) concentrations in rheumatoid arthritis patients dosed orally at 6 mg/kg/day HCQ sulfate for six months. A and B are HCQ concentrations in plasma and blood, respectively. C and D are DHCQ concentrations in plasma and blood, respectively. Dotted lines are 5th and 95th percentiles for prediction intervals. AFE: average fold error; AAFE: absolute average fold error; WPI: proportion within 95% prediction intervals
