# Supplementary material for: A linked physiologically based pharmacokinetic model for hydroxychloroquine and metabolite desethylhydroxychloroquine in SARS‐CoV‐2(−)/(+) populations
Source: Clin Transl Sci. 2023 Apr 29;16(7):1243–57. doi: 10.1111/cts.13527 (PMC10339702; doi:10.1111/cts.13527)
Supplement: Supplementary file 4 — Figure S2 [file CTS-16-1243-s008.pdf]

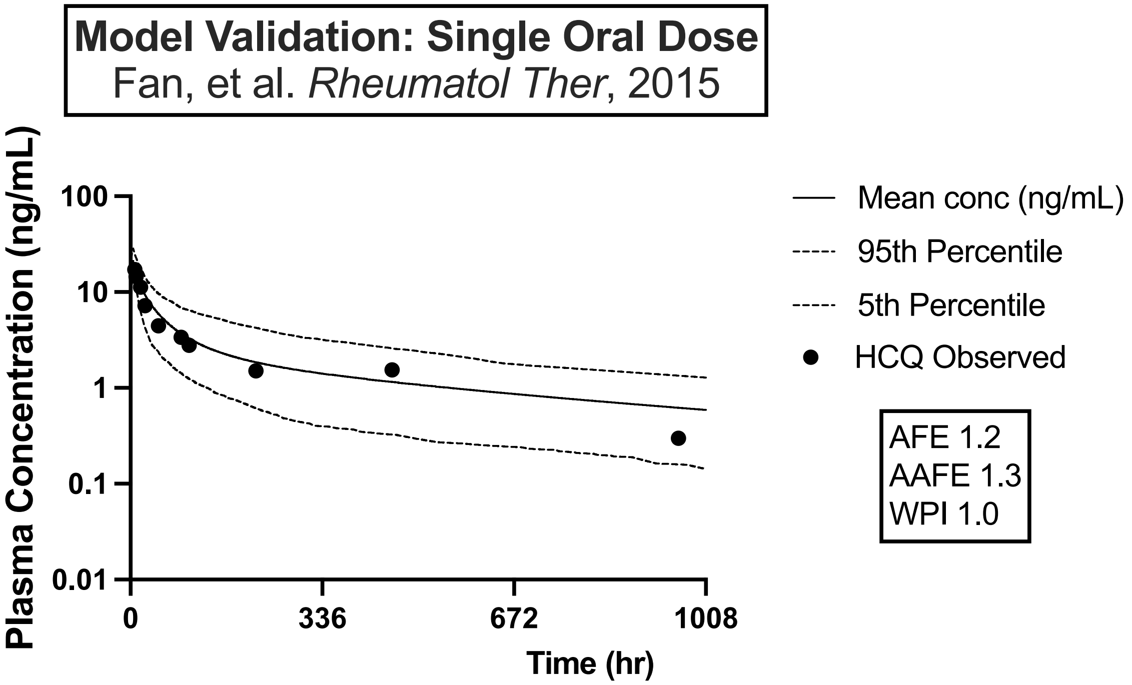

Figure S2: Mean observed (circles) and simulated (solid line) hydroxychloroquine (HCQ) plasma concentrations observed after oral dosing. Healthy volunteers were administered a single 200 mg dose of HCQ sulfate. Dotted lines are 5th and 95th percentiles for prediction intervals. AFE: average fold error; AAFE: absolute average fold error; WPI: proportion within 95% prediction intervals
