# Supplementary material for: A linked physiologically based pharmacokinetic model for hydroxychloroquine and metabolite desethylhydroxychloroquine in SARS‐CoV‐2(−)/(+) populations
Source: Clin Transl Sci. 2023 Apr 29;16(7):1243–57. doi: 10.1111/cts.13527 (PMC10339702; doi:10.1111/cts.13527)
Supplement: Supplementary file 5 — Figure S3 [file CTS-16-1243-s005.pdf]

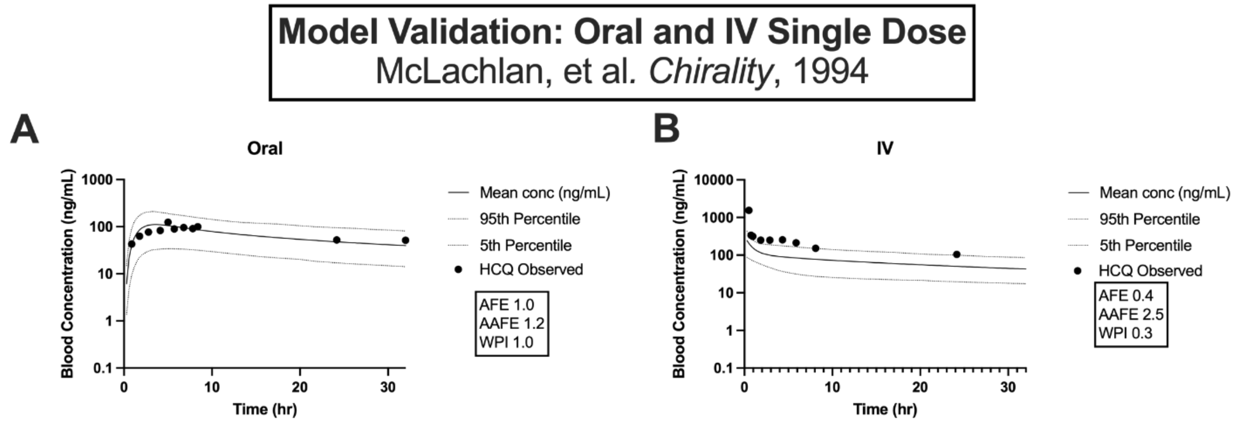

Supplemental Figure 3: Observed (circles) and simulated (solid line) hydroxychloroquine (HCQ) blood concentrations observed after oral (A) and IV (B) dosing in subjects with rheumatoid arthritis. Subjects were administered a single 200 mg dose orally of the HCQ sulfate salt (equivalent to 155 mg base) and a 155 mg HCQ dose IV. Dotted lines are 5th and 95th percentiles for prediction intervals. AFE: average fold error; AAFE: absolute average fold error; WPI: proportion within 95% prediction intervals
