# Supplementary material for: A linked physiologically based pharmacokinetic model for hydroxychloroquine and metabolite desethylhydroxychloroquine in SARS‐CoV‐2(−)/(+) populations
Source: Clin Transl Sci. 2023 Apr 29;16(7):1243–57. doi: 10.1111/cts.13527 (PMC10339702; doi:10.1111/cts.13527)
Supplement: Supplementary file 6 — Figure S4 [file CTS-16-1243-s006.pdf]

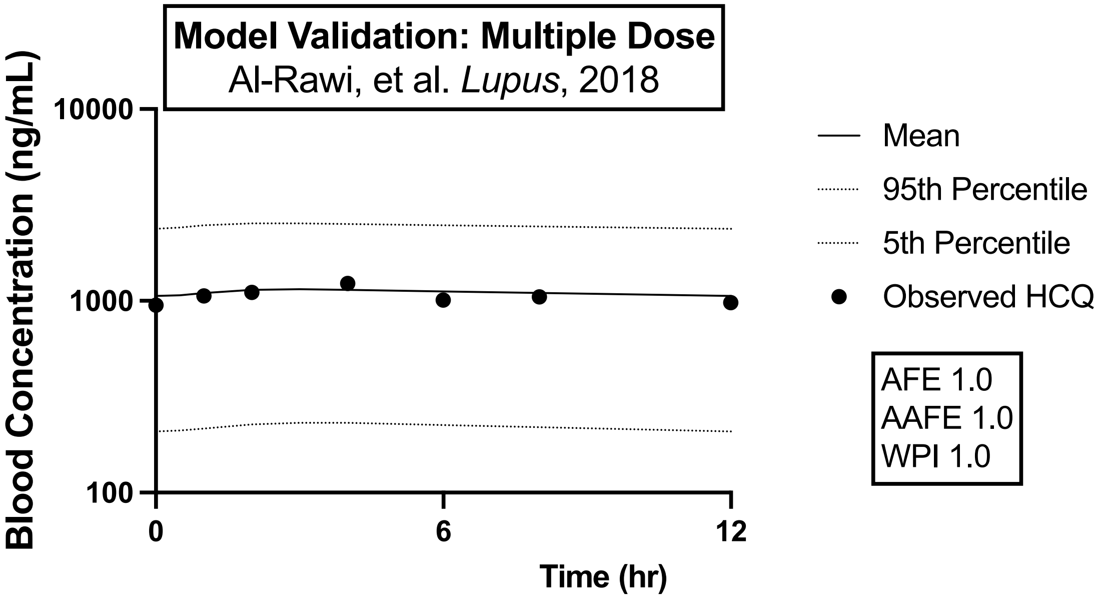

Figure S4: Mean observed (circles) and simulated (solid line) hydroxychloroquine (HCQ) co ncentrations in blood collected from cutaneous lupus patients dosed 200 mg orally with HCQ sulfate twice daily for six months. Dotted lines are 5th and 95th percentiles for prediction intervals. AFE: average fold error; AAFE: absolute average fold error; WPI: proportion within 95% prediction intervals
