# Supplementary material for: A linked physiologically based pharmacokinetic model for hydroxychloroquine and metabolite desethylhydroxychloroquine in SARS‐CoV‐2(−)/(+) populations
Source: Clin Transl Sci. 2023 Apr 29;16(7):1243–57. doi: 10.1111/cts.13527 (PMC10339702; doi:10.1111/cts.13527)
Supplement: Supplementary file 8 — Figure S6 [file CTS-16-1243-s003.pdf]

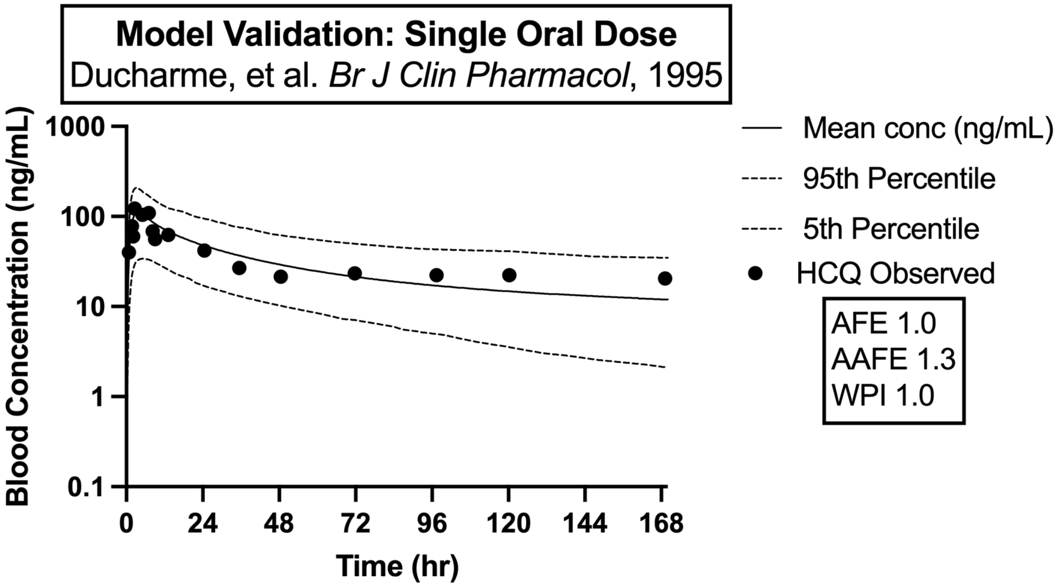

Supplemental Figure 6: Mean observed (circles) and simulated (solid line) blood hydroxychloroquine (HCQ) concentrations in healthy volunteers given a single 200 mg oral dose of HCQ sulfate. Dotted lines are 5th and 95th percentiles for prediction intervals. AFE: average fold error; AAFE: absolute average fold error; WPI: proportion within 95% prediction intervals
