# Supplementary material for: A linked physiologically based pharmacokinetic model for hydroxychloroquine and metabolite desethylhydroxychloroquine in SARS‐CoV‐2(−)/(+) populations
Source: Clin Transl Sci. 2023 Apr 29;16(7):1243–57. doi: 10.1111/cts.13527 (PMC10339702; doi:10.1111/cts.13527)
Supplement: Supplementary file 9 — Figure S7 [file CTS-16-1243-s009.pdf]

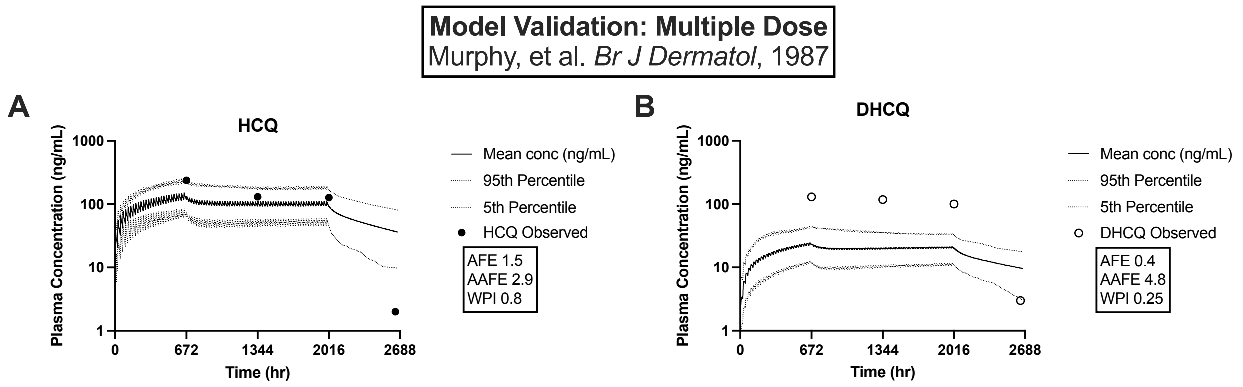

Figure S7: Mean observed (circles) and simulated (solid line) plasma hydroxychloroquin e (HCQ) and desethylhydroxcyhloroquine (DHCQ) concentrations in polymorphic light eruption patients administered 400 mg HCQ sulfate daily for four weeks followed by 200 mg daily for 8 weeks. Dotted lines are 5th and 95th percentiles for prediction intervals. AFE: aver age fold error; AAFE: absolute average fold error; WPI: proportion within 95% prediction in tervals
