# Supplementary material for: A linked physiologically based pharmacokinetic model for hydroxychloroquine and metabolite desethylhydroxychloroquine in SARS‐CoV‐2(−)/(+) populations
Source: Clin Transl Sci. 2023 Apr 29;16(7):1243–57. doi: 10.1111/cts.13527 (PMC10339702; doi:10.1111/cts.13527)
Supplement: Supplementary file 10 — Figure S8 [file CTS-16-1243-s004.pdf]

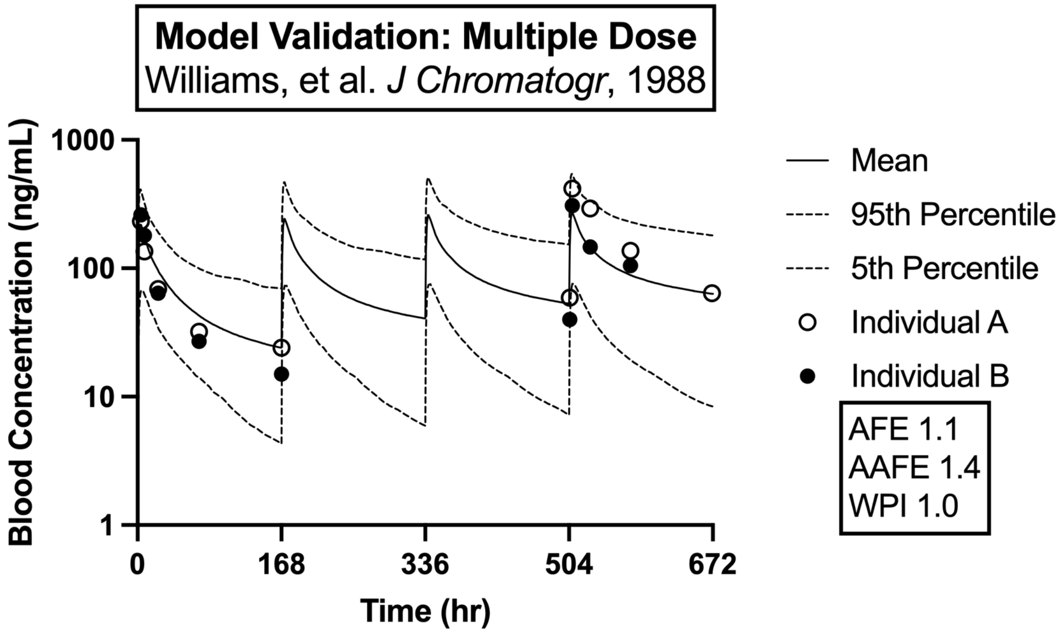

Figure S8: Observed (circles) and simulated (solid line) hydroxychloroquine (HCQ) blood conc entrations in two participants receiving HCQ as prophylaxis for malaria administered 400 mg HCQ sulfate weekly for 4 weeks. Dotted lines are 5th and 95th percentiles for prediction interval s. AFE: average fold error; AAFE: absolute average fold error; WPI: proportion within 95% pre diction intervals
