# Supplementary material for: A linked physiologically based pharmacokinetic model for hydroxychloroquine and metabolite desethylhydroxychloroquine in SARS‐CoV‐2(−)/(+) populations
Source: Clin Transl Sci. 2023 Apr 29;16(7):1243–57. doi: 10.1111/cts.13527 (PMC10339702; doi:10.1111/cts.13527)
Supplement: Supplementary file 11 — Figure S9 [file CTS-16-1243-s007.pdf]

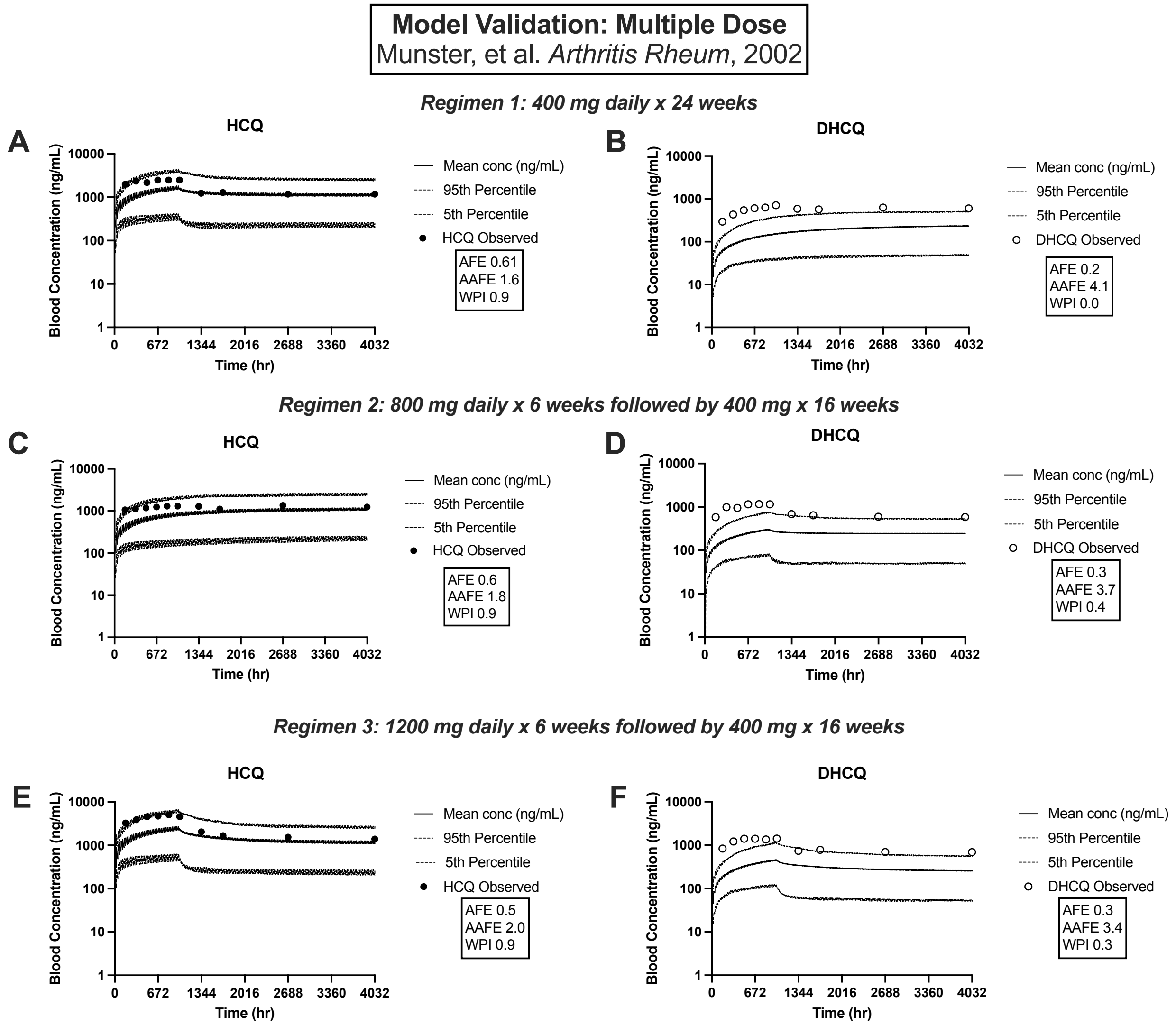

Supplemental Figure 9: Observed (circles) and simulated (solid line) blood hydroxychloroquine (HCQ) and desethylhydroxychloroquine (DHCQ) concentrations in rheumatoid arthritis patients receiving 400 mg orally for 24 weeks (regimen 1), a loading dose of 800 mg daily for 6 weeks followed by 400 mg orally for 18 weeks (regimen 2), and a loading dose of 1200 mg daily for 6 weeks followed by 400 mg orally for 18 weeks (regimen 3). A and B are HCQ and DHCQ blood concentrations after taking regimen 1, respectively. C and D are HCQ and DHCQ concentrations in blood after receiving regimen 2, respectively. Lastly, E and F are HCQ and DHCQ concentrations in blood after receiving regimen 3, respectively. Dotted lines are 5th and 95th percentiles for prediction intervals. AFE: average fold error; AAFE: absolute average fold error; WPI: proportion within 95% prediction intervals
