# Supplementary material for: A linked physiologically based pharmacokinetic model for hydroxychloroquine and metabolite desethylhydroxychloroquine in SARS‐CoV‐2(−)/(+) populations
Source: Clin Transl Sci. 2023 Apr 29;16(7):1243–57. doi: 10.1111/cts.13527 (PMC10339702; doi:10.1111/cts.13527)
Supplement: Supplementary file 12 — Table S3 [file CTS-16-1243-s002.pdf]

Supplemental Table 3: Summary of studies used for model validation.

Supplemental Figure Number	Citation	Subjects	Sampling Matrix	Dose	Frequency	Treatment Length	Route	Analyte	AFE	AAFE	WPI
1	Miller, et al. *DICP, 1991*	Rheumatoid Arthritis Patients	Blood and Plasma	6 mg/kg x 6 months	Daily	Six months	Oral	HCQ Blood	1.6	1.6	1.0
								HCQ Plasma	1.5	1.5	1.0
								DHCQ Blood	0.7	1.4	1.0
								DHCQ Plasma	0.5	1.8	0.1
2	Fan, et al. *Rheumatol Ther,* 2015	Healthy Volunteers	Plasma	200 mg	Single Dose	Single Dose	Oral	HCQ	1.2	1.3	1.0
3	McLachlan, et al. *Chirality*, 1994	Rheumatoid Arthritis	Blood	Oral: 200 mg HCQ sulfate (equivalent to 155 mg base) x 6 months<br>IV: 155 mg	Single Dose	Single Dose	Oral/IV	Oral HCQ	1.0	1.2	1.0
								IV HCQ	0.4	2.5	0.3
4	Al-Rawi, et al. *Lupus*, 2018	Cutaneous Lupus Erythematosus Patients	Blood	200 mg HCQ sulfate (equivalent to 155 mg base)	twice daily	6 months	Oral	HCQ	1.0	1.0	1.0
5	Liu, et al. *Arzneimittelforschung*, 2012	Healthy Volunteers	Blood	200 mg HCQ sulfate (equivalent to 155 mg base)	Single Dose	Single Dose	Oral	HCQ	0.6	1.6	0.9
6	Ducharme, et al. *Br J Clin Pharmacol*, 1995	Healthy Volunteers	Blood	200 mg HCQ sulfate (equivalent to 155 mg base)	Single Dose	Single Dose	Oral	HCQ	1.0	1.3	1.0

7	Murphy, et al. *Br J Dermatol*, 1987	Patients with polymorphous light eruption	Plasma	400 mg HCQ sulfate (equivalent to 310 mg base) 200 mg HCQ sulfate (equivalent to 155 mg base)	Daily	400 mg x 1 month followed by 200 mg x 2 months	Oral	HCQ	1.5	2.9	0.8
								DHCQ	0.4	4.8	0.3
8	Williams, et al. *J Chromatogr*, 1988	Patients receiving Malarial Prophylaxis	Blood	400 mg HCQ sulfate (equivalent to 310 mg base)	Weekly	1 month (4 weeks)	Oral	HCQ	1.1	1.4	1.0
9	Munster, et al. *Arthritis Rheum*, 2002	Rheumatoid Arthritis Patients	Blood	Regimen 1: 400 mg (equivalent to 310 mg base) x 24 weeks Regimen 2: 800 mg (equivalent to 620 mg base) x 6 weeks; 400 mg x 18 weeks Regimen 3: 1200 mg (equivalent to 930 mg base) x 6 weeks; 400 mg x 18 weeks	Daily	24 weeks total	Oral	Regimen 1 HCQ	0.6	1.6	0.9
								Regimen 1 DHCQ	0.2	4.1	0.0
								Regimen 2 HCQ	0.6	1.8	0.9
								Regimen 2 DHCQ	0.3	3.7	0.4
								Regimen 3 HCQ	0.5	2.0	0.9
								Regimen 3 DHCQ	0.3	3.4	0.3
